# The PINCH-Phone: a new screenings method for recurrent incisional hernias

**DOI:** 10.1007/s00464-018-6567-4

**Published:** 2018-11-14

**Authors:** Nadine van Veenendaal, Marijn M. Poelman, Baukje van den Heuvel, Boudewijn J. Dwars, W. Hermien Schreurs, Jan H. M. B. Stoot, H. Jaap Bonjer

**Affiliations:** 1 Department of Surgery, VU University Medical Center, Boelelaan 1117, 1081 HV Amsterdam, The Netherlands; 2 Department of Surgery, Sint Franciscus Gasthuis, Rotterdam, The Netherlands; 3 Department of Surgery, Radboud Ziekenhuis, Nijmegen, The Netherlands; 4 Department of Surgery, Slotervaart Medical Center, Amsterdam, The Netherlands; 5 Department of Surgery, NoordWest Ziekenhuisgroep, Alkmaar, The Netherlands; 6 Department of Surgery, Zuyderland Medical Center, Sittard/Heerlen, The Netherlands

**Keywords:** Incisional hernias, Hernia surgery, Patient-reported outcomes

## Abstract

**Background:**

Debate persists on the optimal management of incisional hernias due to paucity of accurate recurrence rates. Reoperation rates implicate a severe underestimation of the risk of a recurrence. Therefore, long-term postoperative clinic visits allowing physical examination of the abdomen are deemed necessary. However, these are time and costs consuming. Aim of this study was to develop and evaluate a new screenings method for recurrent hernias, the ‘PINCH-Phone’ (Post-INCisional-Hernia repair-Phone).

**Methods:**

The PINCH-Phone is a telephone questionnaire. In this multicenter prospective study, the PINCH-Phone was answered by patients after incisional hernia repair. Afterwards the patients were seen at the clinic and physical examination was done to detect any recurrences.

**Results:**

The PINCH-Phone questions were answered by 210 patients with a median postoperative follow-up of 36 months. Fifty-six patients were seen after multiple incisional hernia repairs. In 137 patients who had replied positively to one or more questions, 28 recurrent incisional hernias were detected at physical examination. Six recurrences were noted in 73 patients who had replied negatively to all questions. The overall sensitivity and specificity of the PINCH-Phone were 82% and 38%, respectively.

**Conclusion:**

The PINCH-Phone appears a simple and valuable screenings method for recurrences after incisional hernia repair and, hence, is recommended for implementation.

Incisional hernias occur in 8–25% after abdominal surgery [1–4]. Patients can experience symptoms such as pain, limitations of daily activities, discomfort, bulging, cosmetic complaints, episodes of incarceration, and reduced health-related quality of life [5–7]. Eighty percent of the patients with incisional hernias undergoes surgical repair [5]. This can be performed either open or laparoscopically [8, 9]. Despite the introduction of the mesh recurrent hernias are still reported in 15–32% of patients after incisional hernia repair [10–13].

Reoperation rates for recurrent incisional hernias have shown to reflect a severe underestimation of the risk of a recurrence [14]. Clinical recurrences account for the majority or the true recurrence rates after incisional hernia repair [4, 13, 15]. Therefore, follow-up of patients after incisional hernia repair is important, but is time consuming and costly. Currently, no routine follow-up after incisional hernia repair exists. It is assumed that patients will report to their physician when symptoms emerge. However, in daily practice patients with recurrences or symptoms might not present themselves, due to unawareness or barriers of visiting their physician [6].

In search of a simple and reliable method of follow-up after incisional hernia repair, we developed a telephone questionnaire to screen for asymptomatic and symptomatic recurrences. Telephone follow-up can be a useful screening tool to monitor outcomes after hernia surgery [16]. Aim of this study was to develop and evaluate a new screenings method for recurrent incisional hernias, the ‘Post-INCisional-Hernia repair-Phone (PINCH-Phone).’ Primary objective was to study the sensitivity, and secondary objective was to study the specificity.

## Materials and methods

### The PINCH-Phone

A telephone questionnaire was developed: Post-INCisional-Hernia-repair-Phone (PINCH-Phone). The questionnaire contains four elements: three questions and self-examination by Valsalva maneuver. The questions of the PINCH-Phone were the following: (1) Do you have any symptoms related to your incisional hernia repair? (2) Have you noticed anything related to your incisional hernia repair? (3) Have you noticed anything related to your incisional hernia repair when coughing, sneezing, or squeezing? (4) Could you please stand up and put one hand flat at the site of your incisional hernia repair? Could you cover your mouth with the other hand and blow? Do you notice anything at the site of your incisional hernia repair?

### Study design

A multicenter prospective study was conducted in patients after incisional hernia repair. Approval by the local ethics committee was obtained in the four participating hospitals: Medical Center Alkmaar, Slotervaart Medical Center, VU University Medical Center, Zuyderland Medical Center Sittard/Heerlen.

All adult patients who had an incisional hernia repair between January 1st, 2012 and December 31st, 2014 were identified by operation code in the hospital databases. Patient files were screened for eligibility for participation. In case of multiple incisional hernia repairs, the most recent repair was considered the index hernia operation. Inclusion criteria were all patients aged over 18 that underwent conventional open or laparoscopic incisional hernia repair between 2012 and 2014. Both primary and recurrent incisional hernia repairs were included, regardless the number of reoperations. All sizes and hernia locations were included. Exclusion criteria were as follows: emergency repair, a history of complex abdominal wall treatment, insufficient understanding of the Dutch language, a mental disorder or the inability to perform physical self-examination. Patients who died or were emigrated were excluded.

Study information brochures and informed consent forms were sent to all patients eligible for the study. After obtaining informed consent, patients were called and the PINCH-Phone was carried out. The answers were recorded and entered into a database. Within 2–4 weeks, patients were seen at the clinic. The researcher was blinded for the answers that were given earlier on the telephone. Subsequently, physical examination was performed to detect recurrences. A clinical recurrence was defined as any abdominal wall gap with or without a bulge in the area of a postoperative scar, palpable or perceptible by clinical examination or imaging [17, 18]. Physical examination was performed both in standing position and laying at the examination table. In both positions, the Valsalva maneuver was conducted. In case of doubt an ultrasound was made.

One researcher carried out all PINCH-Phone questionnaires and performed the physical examination at the clinic. The researcher was independent, not involved with the initial treatment, and not responsible for the health care-related consequences of the outcomes. Primary outcome was detection of a recurrence. Specifics of the hernia and details of the method or repair were obtained from patients’ files.

### Statistical analysis

The aim of this study was to study the sensitivity and specificity of the PINCH-Phone. The required sample size was calculated based on recurrence percentages reported in the literature, and estimated to be 15%. We aimed to calculate the specificity, but especially the sensitivity with a certain reliability, and we therefore needed a minimum of 30 patients with a recurrence. The sample size accordingly is 30/15 × 100 = 200 participants. Considering the risk of drop out, we invited 220 patients. The sensitivity and specificity of the PINCH-Phone as a diagnostic tool were calculated by comparing its outcomes with the outcomes of the golden standard for detecting clinical recurrences, clinical examination.

The 95% confidence intervals were calculated. For all statistical procedures, a probability value (*p* value) < 0.05 was considered to be statistically significant. Analysis of data was performed in SPSS version 2.0.

## Results

Medical records of 779 patients who had an operation code for ‘incisional hernia repair’ in 2012–2014 in the four participating centers were screened. After exclusion of the deceased and other exclusion criteria, 621 patients were eligible for the study.

621 patients were sent patient trial information, of which 240 (39%) patients returned the informed consent forms. Nineteen patients could not be reached by telephone, despite repeatedly trying. The PINCH-Phone was carried out in 221 patients, and all were scheduled for clinical visit. Executing the PINCH-Phone took approximately 3 min.

**Fig. 1:**
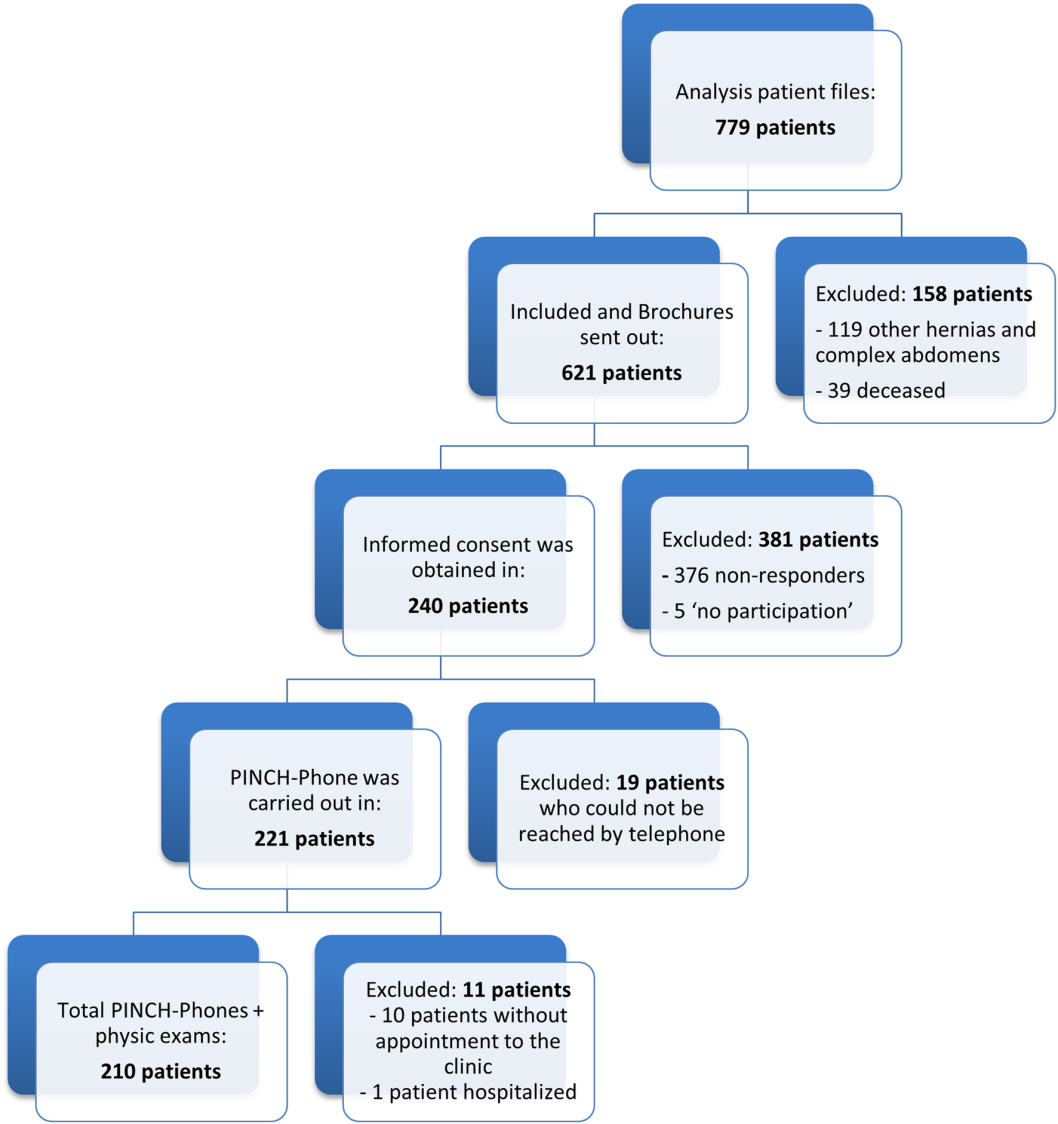
Flowchart of the study

A total of 210 patients showed up for clinical visit. Due to logistical problems, ten patients cancelled their appointment. One patient was hospitalized between the PINCH-Phone and clinical visit due to a mesh infection. All participants were included in a study period of 12 months, from December 2015 till December 2016. A flow diagram of study enrollment is shown in Fig. 1.

Patient characteristics, hernia details and surgery techniques are given in Table 1. Two hundred and ten patients were included in the study. The population consisted of 105 (50%) males, and 105 (50%) females. The median age at operation was 58.4 (range 20–93) years and BMI 29.8 (range 17.7–53.1). 154 (73%) patients were seen after primary incisional hernia repair and 56 (27%) patients were seen after multiple repairs. Hernia defects varied from 0.5 till 22 cm as longest diameter. The mean interval between index incisional hernia repair and enrollment in the study was 36 (range 12–49) months. Ninety-nine (47.1%) patients had undergone laparoscopic incisional hernia repair, and 111 (52.9%) patients had undergone conventional open repair. In the open repair group, 94 patients underwent mesh repair and 17 patients non-mesh suture repair.

**Table 1:** Patient and hernia characteristics, follow-up and surgical technique

	Incisional hernias (*n* = 210)
Female:male	105:105
Age (years)	58 (20–93)
BMI	29.8 (18–53)
Follow-up (mo)	36 (12–49)
Defect size (cm)	6 (0.5–22)
Primary:recurrent hernias	154:56
Surgical technique	
Laparoscopic	99 (47%)
Open mesh repair	94 (45%)
Open suture repair	17 (8%)

Data are given in numbers (percentages) and median (range) when relevant

The first question of the PINCH-Phone concerned the presence of symptoms related to their incisional hernia repair. Eighty-nine patients experienced symptoms, such as pain, discomfort or a lump. Twenty patients had a recurrence. One-hundred-twenty-one patients had no complaints of their repaired incisional hernia, of which 14 patients had a recurrence (Table 2). The sensitivity of this question was 58.8% (95% CI 41–75) and the specificity was 92% (95 CI 53–68).

**Table 2:** Results of the PINCH-Phone questions

PINCH-Phone	Physical examination (PE)		
	Recurrence at PE	No recurrence at PE	Total
Ql: symptoms related to surgical site			
Symptoms	20 (10%)	69 (33%)	89 (42%)
No symptoms	14 (7%)	107 (50%)	121 (58%)
Q2: signs related to surgical site			
Noticed something	22 (10%)	64 (30%)	86 (41%)
Noticed nothing	12 (6%)	112 (53%)	124 (59%)
Q3: signs during sneezing, squeezing or coughing			
Noticed anything by ↑ pressure	15 (7%)	38 (18%)	53 (25%)
Noticed nothing by ↑ pressure	19 (9%)	138 (66%)	156 (75%)
Q4: self-examination with Valsalva maneuver by patient			
Swelling at Valsalva	20 (10%)	42 (20%)	62 (30%)
No swelling at Valsalva	14 (7%)	134 (63%)	148 (70%)
Overall outcomes PINCH-Phone^a^			
Positive	28 (13%)	109 (52%)	137 (65%)
Negative 6 (3%)	67 (32%)	73 (35%)	
Total	34 (16%)	176 (84%)	210

^a^Minimum of one PINCH-Phone question answered positively by the patient

Secondly, patients were asked whether they had noticed anything related to their incisional hernia repair. Eighty-six patients had noticed something, of which 22 patients had a recurrence. One hundred and twenty-four patients had no complaints of their repaired incisional hernia, of which 12 patients had a recurrence (Table 2). The sensitivity of this question was 64.7% (95 CI 46–80) and the specificity was 63.6% (95 CI 56–71).

The third question referred to whether patients had noticed anything at the location of the former incisional hernia during increased abdominal pressure, such as coughing, sneezing or squeezing. Fifty-three patients had noticed something during elevated abdominal pressure, of which 15 patients had a recurrence. One hundred and fifty-seven patients had noticed nothing, of which 19 patients had a recurrence (Table 2). The sensitivity of this question was 44.1% (95% CI 28–62) and the specificity was 78.4% (95% CI 71–84).

The last, fourth part of the PINCH-Phone was self-examination by Valsalva maneuver. Sixty-two patients had noticed something during the Valsalva maneuver, of which 20 patients had a recurrence. One hundred and forty-eight patients felt nothing during self-examination, of which 14 patients had a recurrence (Table 2). The sensitivity of this question was 58.8% (95% CI 41–75) and the specificity was 76.1% (95% CI 69–82).

In total, 34 recurrences (16%) were found at physical examination: 14 in the open group and 20 in the laparoscopic group. Twenty-eight patients had responded positively to at least one PINCH-Phone question. Six patients had answered ‘no’ to all PINCH-Phone questions, but had a recurrence at physical examination. Fifty-four patients that underwent laparoscopic incisional hernia repair suffered from bulging. No ultrasounds were conducted to diagnose recurrences.

Overall, 137 patients answered ‘yes’ to at least one PINCH-Phone question, of which 28 patients had a clinical recurrence. 73 patients answered ‘no’ to all questions, of which six patients were diagnosed with a recurrence (Table 2). Sensitivity of the PINCH-Phone was 82.4% (95% CI 65–93) and specificity was 38.1% (95% CI 31–46). The positive predictive value was 20.4% (95% CI 14–28) and the negative predictive value was 91.8% (95% CI 82–97).

None of the PINCH-Phone questions individually could be considered as best discriminating question. Therefore, we analyzed whether a combination of questions offered any additional value. Whereas sensitivity became less when adding more questions, specificity increased up to 0.92 for all four PINCH-Phone questions.

## Discussion

In our study, 137 (65%) patients had a positive PINCH-Phone, of which 28 patients had a recurrence. Our findings show an overall sensitivity of the PINCH-Phone of 82,4%. Twenty percent (28/137) of the patients with a positive PINCH-Phone had a recurrence. This means that 51% (109/210) of all patients after incisional hernia repair will be invited to the clinic to exclude a recurrence. A visit to the clinic in this population is justified though, because all these patients responded positively to one or more PINCH-Phone questions. Evaluation of the symptoms at the clinic is required. Thirteen percent (28/210) of the patients will be rightly invited to confirm a recurrence at the clinic.

Unfortunately not all recurrences were detected by the PINCH-Phone. Seventy-three (35%) patients responded negatively to all PINCH-Phone questions, of which six patients had a clinical recurrence. The specificity of the PINCH-Phone is 38.1%. If a patient has a negative PINCH-Phone a recurrence cannot be excluded with certainty. Our results show that 3 (6/210) percent of the patients will not be invited to the clinic and that a recurrence will be missed. We consider this small percentage as acceptable. These patients had no symptoms and therefore one might suggest that there is no clinical evidence. Use of the PINCH-Phone shows that in 32% (67/210) of the cases a visit to the clinic is not necessary and can be prevented.

Although the PINCH-Phone did not detect all recurrences, 82% recurrences were detected. Eighteen percent of the recurrences were missed, lacking any symptoms. The PINCH-Phone can prevent unnecessary visits to the hospital in one-third of the patients after incisional hernia repair. The PINCH-Phone as a screening method will result in an invitation to the clinic in 51% of the patients after hernia surgery. Therefore, the PINCH-Phone can be used as a simple first screening method in the follow-up of patients after incisional hernia repair.

In our study, both primary and recurrent incisional hernias were included. A quarter of the study group had a recurrent incisional hernia, which have higher recurrence rates than primary incisional hernias [19]. Since the main objective of this study was the sensitivity of the PINCH-Phone and not hernia characteristics, subanalyses regarding hernia size and location were not made. For future studies larger cohorts with detailed registration of patient and hernia characteristics are needed to investigate whether these aspects are correlated with recurrence after incisional hernia repair.

Recurrences are a common complication after incisional hernia repair [11], leading to general problems within the physical and emotional domains. For surgeons and patients, it is of great importance to have an adequate follow-up to detect symptoms and recurrent hernias in an early stage. Methods advocated to analyze the outcome of hernia repair include clinical examination, postal questionnaires, telephone interviews or a combination [24]. Since clinical follow-up is time consuming for both patient and doctor, questionnaires have been proposed as an alternative method of follow-up [16].

Previous studies have aimed to validate follow-up questionnaires after hernia repair. Written questionnaires were developed for follow-up after inguinal hernia repair, but resulted in high false-positive and false-negative rates [16, 20–22]. Vos et al. showed that approximately half of the recurrences would be missed with the questionnaire only and concluded that physical examination is the only reliable method for evaluating the quality of hernia surgery [21]. Defining recurrence rates by reoperation rates is known to underestimate true recurrence rates in hernia surgery [14, 22, 23].

The use of questionnaires in detecting incisional hernias has been described less intensively. Earlier studies showed that patients with feelings of discomfort at the site of their scar have a high risk on the presence of an incisional hernia [24]. Luijendijk et al. showed that patient-perceived feelings of a recurrence were predictive for an actual recurrence [8]. Baucom et al. developed the Ventral Hernia Recurrence Inventory to detect recurrent incisional hernias. Although the three questions of this algorithm seem very promising, the study population was very small and had a potential selection bias [25]. Our PINCH-Phone consists of a bigger study population and is therefore more reliable to draw conclusions on the predictive value of a questionnaire.

Currently, surgical meshes are classified as group II medical devices under regulations of the US Food and Drug Administration (FDA) and the EU Medical Device Directive (CE mark) [26]. Due to several adverse events with surgical meshes [27] and discrepancies between premarket animal studies and clinical studies [28], concerns have been expressed about the safety of surgical meshes [29]. In order to improve overall safety of medical devices on the EU market, surgical meshes are up-classified to class III devices by the European Commission in the new EU regulation [30, 31]. As of 2020, these new regulations will entry into force, making registry and follow-up of patients after mesh repair even more relevant. The PINCH-Phone can contribute in the follow-up of patients to screen for recurrences.

A remarkable secondary finding in this study is the large proportion of patients who reported symptoms related to the site of their operation (89/210). This is higher than the 20–23% of patients that reported pain at the site of their scar in earlier studies [11]. Our percentage, however, is comparable to the 69% experiencing pain in the study by Baucom et al. In this study, a large proportion of patients reported sporadically (69%) or actively (48%) pain or symptoms at the site of their operation [25]. In the last two decades there has been an increased interest in quality of life in surgical research [32]. Recently, patient-reported outcomes have gained popularity in incisional hernia research as well [33–39]. In our study, 89 patients reported positively to the first PINCH-Phone question concerning symptoms. The outcomes of this study offer perspectives for future research with relevance for patient-reported outcomes in incisional hernia repair.

### Limitations

The results of this study should be considered in light of several limitations. The first limitation in this study is the large group of nonresponders. Information brochures and informed consent forms were sent to 621 patients of which 381 (61%) people did not respond. Assumably, these patients either did not receive the mail, were not willing to participate or forgot to send back the informed consent form. Nonresponse is a known disadvantage of postal questionnaires in research. Strategies to improve the response rate, such as reminder letters and telephone contact, have proven to be effective ways to improve response rates [16]. In our study 39% responded, which is in keeping with a questionnaire answer rate that varies from 25 to 30% when no follow-up or reminder is sent [40, 41]. Since the required sample size was reached within the study period reminders were not sent, leading to a potential selection bias. Patients that suffer from complaints are more likely to participate than those who do not have complaints at all [25].

Another limitation of this study is that all recurrences were detected by physical examination. There are no clear diagnostic criteria for incisional hernias and guidelines have no consensus on this [42]. Studies have shown that neither physical examination nor ultrasonography have a sensitivity and specificity of 100% [43]. Recurrences are correctly diagnosed in 88% of the cases by physical exam. Therefore, we decided to perform physical examination first, and perform ultrasonography in case of doubt.

In our study, both primary and recurrent incisional hernias were included, which can cause potential selection bias. A quarter of the study group underwent multiple repairs, which is comparable to other studies [44]. The variation in time of follow-up after incisional hernia repair can be considered a limitation of the study. Recurrences can occur 10 years after repair, so patients can still develop a recurrence after participation of the study. Since recurrences develop mainly in the first year after repair, we assume that we have included the majority of incisional hernias [45]. A subgroup analysis for surgical technique was also beyond the scope of this study. Since the main objective of this study was the sensitivity of the PINCH-Phone and not hernia characteristics, subanalyses regarding hernia size and location were not made. For future studies larger cohorts with detailed registration of patient and hernia characteristics and surgical technique are suggested to investigate whether these aspects are correlated with recurrence after incisional hernia repair.

## Conclusion

In light of persisting high recurrence rates and the new European legislation for surgical devices, we aimed for improvement of follow-up of patients after incisional hernia repair. We developed a telephonic questionnaire ‘the PINCH-Phone,’ containing three questions and a do-it-yourself Valsalva maneuver. The PINCH-Phone had a sensitivity of 82% and can be considered a simple and reliable screenings method for recurrences and, hence, is recommended for implementation. Additionally, the outcomes of this study highlight the need for increased attention for patient-reported outcomes after incisional hernia repair.
